# Respiratory Infections in the Aging Lung: Implications for Diagnosis, Therapy, and Prevention

**DOI:** 10.14336/AD.2023.0329

**Published:** 2023-08-01

**Authors:** Antje Häder, Nilay Köse-Vogel, Luise Schulz, Lucja Mlynska, Franziska Hornung, Stefan Hagel, Ulf Teichgräber, Susanne M Lang, Mathias W Pletz, Claude Jourdan Le Saux, Bettina Löffler, Stefanie Deinhardt-Emmer

**Affiliations:** ^1^Institute of Medical Microbiology, Jena University Hospital, Jena, Germany.; ^2^Department of Radiology, Jena University Hospital, Jena, Germany.; ^3^Institute of Infectious Diseases and Infection Control, Jena University Hospital—Friedrich Schiller University Jena, Jena, Germany.; ^4^Clinic for Internal Medicine V, Pneumology, Jena University Hospital, Jena, Germany.; ^5^Medicine/Pulmonary and Critical Care Division, University of California San Francisco, San Francisco, California, USA.; ^6^Leibniz Institute of Photonic Technology - Member of the research alliance “Leibniz Health Technologies”, Albert-Einstein-Straße 9, Jena, Germany.

**Keywords:** Aging, lung, pneumonia, microbiological diagnostic, radiological procedures, therapy, vaccination

## Abstract

Respiratory infections pose a significant health problem among elderly individuals, particularly during the COVID-19 pandemic. The increased mortality and morbidity rates among individuals over 65 highlight the criticality of these infections. The normal aging process in the lungs increases vulnerability to respiratory infections due to the accumulation of cellular damage and senescence. Consequently, the lung environment undergoes major changes in mechanical function and other systemic factors. This review aims to examine the influence of aging on respiratory infections from a clinical perspective by analyzing clinical studies. Additionally, the review will emphasize potential prevention and diagnostic developments to enhance therapy options available for elderly patients over 65 years of age.

## 1. Introduction

Respiratory infections are among the leading causes of death worldwide, affecting in particular people over 65 years due to the severities of the infections experienced by this demographic [1].

The burden of respiratory infections in the elderly population has been highlighted recently during the Coronavirus Disease 2019 (COVID-19) pandemic, with increased mortality and morbidity rates in this susceptible population [2-4]. In addition, influenza virus epidemics and pandemics also highlight age as a crucial risk factor for severe infections [5]

Several physiological changes in the elderly have been noted as risk factors for pneumonia. Structural changes in lung physiology over the course of normal aging leave older individuals with less ability to cope with infections of the lungs [6]. It is well recognized that the presence of a lung disease, any comorbidity, and age-related changes in the immune system all increase the risk of pneumonia [6-8].

Interestingly, symptoms of pneumonia are less typical in the elderly, e.g., new onset confusion and less frequently fever, due to age-related changes in the immune response [9, 10]. This leads to a delayed diagnosis and therefore to a deferred start to therapy, prolonged hospital stays, higher mortality rates, and even further nosocomial spread of the disease [11].

Therefore, it is essential to understand the burden of respiratory infections have on the elderly, to address the clinical need for improved diagnostic approaches and treatment strategies.

This review describes the structural changes in the aging lung, emphasizing the reasons underlying the increased susceptibility of the elderly to respiratory infections. We aim to investigate the influence of aging on respiratory infections from a clinical perspective. For this purpose, we discuss clinical studies and highlight potential diagnostic and therapeutic improvements to support the clinical management of patients over 65 years of age.

## 2. Structural changes in the aging lungs

Aging is defined as the time-dependent accumulation of cellular damage resulting in gradual decline in tissue function and increased susceptibility to disease and death [12]. The nine hallmarks of aging, i.e.: genomic instability, telomere attrition, epigenetic alterations, loss of proteostasis, deregulated nutrient sensing, mitochondrial dysfunction, cellular senescence, stem cell exhaustion, and altered intercellular communication; have already been defined in the aging lung, with an additional proposed hallmark, extracellular matrix (ECM) dysregulation [13-15]. These ten hallmarks of the aging lung are all also associated with the pathogenesis of lung-diseases in the elderly [14, 15].

During the process of aging, the lung environment and mechanical function drastically modify breathing and vulnerability to infections [16]. The main alteration to lung function is the loss of lung elasticity with age. Lungs become more rigid due to a feedback loop altering the expression of ECM proteins [17]. Changes in the collagen fiber network lead to alveolar duct dilatation and enlargement of alveolar air spaces, which contribute to decreased alveolar surface tension and lung compliance [18]. Additionally, respiratory muscle strength and performance also decline, becoming less able to contribute to ventilation and resulting in loss of compliance of the chest wall [19]. Decreased expiratory and inspiratory strengths lead to impaired respiratory mechanics. Furthermore, the thoracic cage stiffens from calcification of the rib cage and loses its ability to expand during inhalation due to age-related kyphosis - in other words, curvature of the spine [20]. Mucocilliary clearance function also becomes impaired, and less efficient clearance of secretion by the mucocilliary system in the aging lung is correlated with pneumonia [21] These age-related changes in the lung structure are unlikely to lead directly to clinically significant diseases in healthy elderly individuals; however they may have substantial impacts when the individual is compromised or dealing with a stressor such as a lower respiratory tract infection.

The lung is composed of more than 40 different cell types, characterized recently in detail by single-cell molecular profiling [22, 23]. The air that is encountered by the lungs contains a long list of deleterious elements that can cause changes to cellular composition and function over time. The main problem for aging lungs is its ineffective tissue repair and remodeling, which are normally orchestrated by epithelial cells, fibroblasts, immune cells, and progenitor cells - all of which undergo age-related functional changes. For instance, in an aged lung, alveolar epithelial type-2 cells (AEC2) lose their regenerative potential. As progenitor cells, the loss of AEC2 leads to a decrease in the numbers of alveolar epithelial type-1 cells (AEC1) [24]. These changes to the alveolar environment favor fibroblast proliferation, and subsequent remodeling leads to airway expansion [25].

The number of senescent cells accumulates with age in the lung, and this process of senescence affects each of these different cells, progressively altering the local environment via senescent-associated secretory phenotype (SASP) and thereby leading to functional changes in neighboring cells [26]. Although the role of senescence in the aging lung is not yet fully described, fibroblast senescence has been associated with pulmonary remodeling and has been linked to age-associated pulmonary diseases such as pulmonary fibrosis [13, 27]. Senescent cells that accumulate with age have also been described to contribute to infection susceptibility in the elderly [28]. Cellular senescence increases the expression of bacterial ligands in the lungs and is positively correlated with increased susceptibility to pneumococcal pneumonia. [28].

## 3. Respiratory infections in the aging lung

Infections of the respiratory system can be classified into upper and lower respiratory infections. The majority of upper respiratory tract infections are of viral origin, whereas lower respiratory tract infections can be caused by either viruses or bacteria. In general, most cases of bronchitis and bronchiolitis are caused by viruses [29].

Pneumonia, one of the leading causes of death in the elderly, is defined as a severe form of acute lower respiratory tract infection which causes acute inflammation of the pulmonary parenchyma in either one or both lungs [30]. Based on how it is acquired, pneumonia can be classified mainly into two groups: pneumonias acquired in a community setting (i.e. community-acquired pneumonia (CAP and hospital-acquired pneumonia (HAP) and ventilator-associated pneumonia (VAP)) [31]. HAP is defined as pneumonia which manifests 48 h or more after admission to hospital, and VAP occurs more than 48 h after endotracheal intubation. CAP occurs 48 h before hospitalization, and HCAP occurs in patients who have frequent contact with the health system, take various antibiotics, and/or are in functional states of frailty [31].

**Figure 1.**
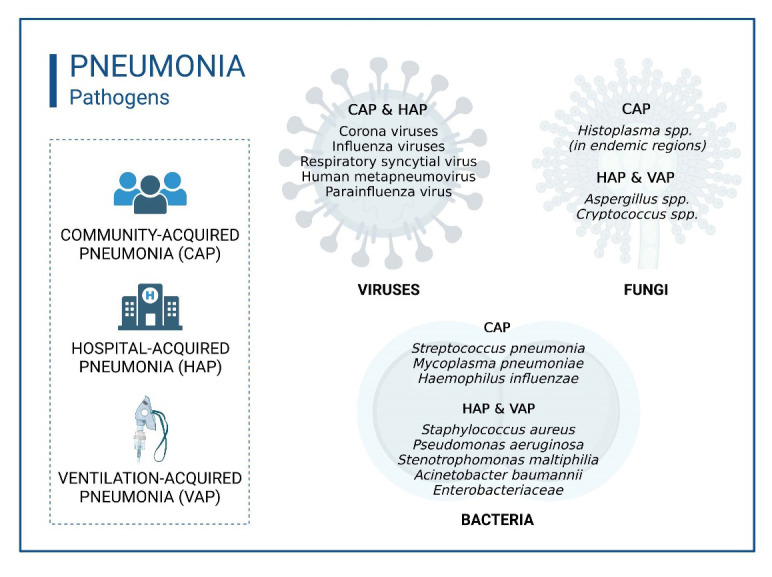
**Overview of respiratory pathogens leading to pneumonia**. Bacterial pneumonia can be divided into community-acquired pneumonia (CAP) and hospital-acquired pneumonia (HAP) according to the pathogen that causes it. Viral pathogens can lead to both CAP and HAP. Fungal pneumonia is predominantly hospital-acquired (HAP). Figure shows a selection of pathogens, not a complete list.

In the 2005 guideline a third entity, healthcare-associated pneumonia (HCAP), was defined by the American Thoracic Society Infectious Diseases Society of America [32]. The idea behind this definition was, that e.g. nursing-home acquired pneumonia (NHAP) exhibits a similar pathogen spectrum with an increased risk for *Pseudomonas aeruginosa* and multi-drug-resistant pathogens like HAP and should be treated as like. However, numerous studies have later shown that this assumption was not valid and has led to a substantial empiric overtreatment of nursing home patients [33-35]. Therefore, the definition HCAP, that was rarely adopted by other scientific societies was later given up. Pneumonias acquired in nursing homes (NHAP) represent an important subgroup of CAP. Although they have only slightly altered pathogen patterns, they differ fundamentally in terms of age, functionality (self-care and self-determination), comorbidity, severity of pneumonia, and prognosis [36]. The highest lethality rate for pneumonia infection is in bedridden patients with NHAP [37]. The classification scheme shown in Figure 1 reflects the variety of pathogens responsible for each infection type and forms the basis for treatment options. Pneumonia is caused by various infectious agents such as bacteria, viruses, and fungi (Fig. 1). CAP is more likely to be caused by bacteria susceptible to first-line antibiotics, or infection by certain pathogens (e. g., influenza virus, *Streptococcus (S.) pneumoniae*) [38]. By contrast, HAP and VAP are more likely to be caused by difficult to treat pathogens such as Pseudomonas aeruginosa or antibiotic-resistant bacteria such as methicillin-resistant *Staphylococcus aureus* (MRSA) and MDR gram-negative bacteria [39, 40].

Independent of the site of acquisition (i.e., community versus hospital-acquired), a third pneumonia entity is defined in recent guidelines, i.e., pneumonia in the immunocompromised host. This entity is defined by an increased risk of opportunistic pathogens, e.g., fungi, reactivation of herpes viruses, non-tuberculous mycobacteria or nocardia or parasites. The spectrum of opportunistic infections is in part shaped by the underlying immunosuppression, e.g., *Pneumocystis jirovecii* pneumonia is typical for T-cell disturbances as HIV and *Aspergillus fumigatus* is a feared infection in the neutropenic host. To date, there is not a comprehensive guideline on pneumonia in the immunocompromised host, but recently and international consensus has been published [41]. The tripartite division of pneumonias into community-acquired, hospital-acquired and pneumonia in the immunocompromised host is also referred as the “pneumonia triad” [42].

Elderly individuals are more susceptible to lung infections, mainly due to changes in the immune system and emerging comorbidities. Defined as the possibility of experiencing a health risk, the susceptibility to infectious diseases is for elderly patients much higher. Additionally, the elderly tends to experience more severe disease, which can also be attributed to impaired immune function, comorbidities, and decreased respiratory reserve.

Both, susceptibility, and severity mainly depend on one of the most important biological systems facing the effects of aging: the immune system. Resulting in an increased susceptibility to infectious diseases and a decreased effectiveness of vaccinations, the immune system undergoes a decline in the composition and function of immune cells [43, 44]. Defined as immunosenescence, the key aspects of the aging immune system include low-level inflammation, increased incidence of autoimmunity, decreased ability to fight infection or cancer and impaired ability to respond effectively to new antigens [45]. Overall, immunosenescence results in an increase of age-related diseases, including neurodegenerative diseases, cancer, cardiovascular diseases, and infectious diseases [44]. The

immune related changes can affect both the innate and adaptive immune system [9]. The age-related changes in immune function are due to a combination of cellular aging and the effect of the aging environment on proliferation and differentiation in response to antigenic stimulation. Innate immune cells contribute to inflammation by producing cytokines associated with chronic inflammation. However, important functions of innate immune cells such as phagocytosis, antigen uptake and presentation, migration, and bactericidal activity decline with age [46].

Additionally, the adaptive immunity, specifically CD4+, CD8+ T-cells, and B cells show age-related functional changes. The B cell function is significantly affected by aging and is associated with decreased class-switch recombination and somatic hypermutation of immunoglobulin genes, which lead to decreased antibody production and function after vaccination or infection [44]. Age-related functional changes include decreased proliferative capacity, inappropriate differentiation of the T-helper subset, and an increase in the proportion of regulatory T-cells [46]. The accumulation of these differentiated CD8+ T-cells leads to a constriction of the immune repertoire and is associated with the reduced immune response to vaccination and novel infections such as SARS-CoV-2 [46].

In conclusion age-related changes in the innate and adaptive immune function increase the susceptibility and the severity of infections such as influenza, COVID-19 and bacterial pneumonia in the elderly compared to younger individuals.

### Bacterial infections

Bacterial pneumonia is one of the major infectious diseases in terms of incidence, mortality, effect on quality of life, and overall impact on society. Although the exact proportions of its bacterial etiology depend on the studied population, CAP has been most frequently associated with *S. pneumoniae,* but also with *Haemophilus (H.) influenzae* and with some atypical organisms such as *Mycoplasma (M.) pneumonia, and Legionella (L.) pneumophila,* while HAP and VAP have mostly been associated with strains of *Staphylococcus (S.) aureus* and gram-negative bacilli such as *Klebsiella (K.) pneumoniae* or *Pseudomonas (P.) aeruginosa* [47-49]. However, studies have shown that while the frequency of pathogens did not differ much between age groups (< 65 years vs ≥ 65 years), *S. pneumoniae* (≥ 65 years: 19.9-85.0 %) and *H. influenzae* (≥ 65 years: 2.9-29.4 %) were found most frequently in elderly patients’ samples, while *M. pneumoniae* was less frequently isolated in the elderly (≥ 65 years: 0.7-6.8 %) [50].

Immunosenescence, or the immunological changes occurring with age, involves decreased efficiency of the immune response, leading to increased susceptibility of elder patients to several infectious diseases such as pneumonia [9, 51]. Immunological changes frequently lead to misdiagnosis in elderly patients with CAP because of specific missing specific symptoms typical of the disease, such as fever, chest pain, or cough. It has been shown that *S. pneumoniae* can bind more efficiently to senescent lung cells due to elevated cellular proteins required for cell entry [28]; however, the inflammatory signaling and the ability of neutrophils to kill *S. pneumoniae* was suppressed in the lungs of aged mice despite higher bacterial burdens [52, 53]. This increased affinity to bind bacteria and higher bacterial burden with suppressed inflammation in the lungs is thought to contribute to the slower rate of infection resolution in the elderly and provides a molecular mechanism for the increased incidence of CAP in the elderly population.

### Viral infections

CAP occurs frequently in infants due to their immature immunological memory. Although viral infections in the elderly are mostly reinfections, the risk of developing pneumonia is increased due to immunosenescence and high-risk conditions such as immunosuppression and chronic diseases [54-56]. Seasonal viral pneumonia occurs mainly during cold fall and winter seasons, from infections of corona viruses, influenza viruses, respiratory syncytial virus (RSV), human metapneumovirus, and parainfluenza viruses.

HAP has an increased mortality rate in the ≥ 65-year age group. The risk is higher in patients with hematological/oncological diseases and after organ transplantation [57]. Nosocomial respiratory viral infections are most frequently induced by influenza A viruses, followed by RSV, parainfluenza viruses, and human metapneumovirus. The virus type has no influence on morbidity and mortality [57-59].

#### Influenza viruses

Influenza viruses cause illness in all age groups, but people ≥ 65 years of age are particularly susceptible to severe disease progression and more at-risk in terms of hospitalization and mortality [60, 61].

Seasonal influenza occurs annually between November and April in the northern hemisphere, and circulating viral strains are surveilled to provide an annually-adjusted vaccine. In contrast, pandemic outbreaks are unpredictable and rare. They often derive from the avian reservoir and disseminate rapidly in the immunologically naïve population, where they can lead to serious clinical and economic consequences [62, 63].

Older people have been at risk of influenza infection throughout their lives. Due to the rapid antigen shift and drift of seasonal influenza viruses, they do not build up lasting immunity to these strains [64]. During seasonal influenza individuals aged ≥ 65 have the highest risks of hospitalization and death [65]. In contrast, during the last pandemic in 2009 due to influenza A (H1N1; “swine flu”), more than 80% of deaths occurred in younger individuals ≤ 65 years [66].

Of all seasonal influenza-associated deaths, more than two-thirds occurred in patients aged ≥ 65, with a mortality rate that is 26% higher compared to younger patients [60]. The World Health Organization (WHO) recommends annual vaccination against seasonal influenza for individuals ≥ 65 years of age. In the US, vaccination is recommended starting at age 50, as about one-third of 50-64-year-olds are high-risk [67]. Comorbidities such as diabetes mellitus, chronic lung disease, cardiovascular diseases, immunosuppression, or renal disease increase the risk of a severe course of the infection [68]. In elderly patients with high-risk conditions, influenza-associated pneumonia causes significant physical limitations and can cause cardiovascular (i.e. myocardial infarction and stroke) and pulmonary complications such as pneumonia or superinfection with bacteria/fungi [65].

A recent review showed that influenza disproportionately burdened the elderly population, with the risk of death nearly doubled in those aged 75 years or over compared to those aged 65-74. Likewise, the risk of hospitalization, rates of emergency department visits, and intensive care unit attendance are highest among the elderly [69]. Moreover, secondary bacterial infections after an influenza infection account for 75% of cases that present as severe pneumonia [70].

#### SARS-CoV-2

The World Health Organization (WHO) reported almost 630 million confirmed COVID-19 cases and over 6.5 million deaths, globally, by October 31th, 2022 [71]. COVID-19 can be asymptomatic or be accompanied by moderate illness, but can also be severe and lead to hospitalization, pneumonia, acute respiratory distress syndrome (ARDS), cardiovascular complications, kidney injury, stroke, ICU admission, and death [72, 73].

Age is a major risk factor for an infection with severe acute respiratory syndrome coronavirus type 2 (SARS-CoV-2) and COVID-19-associated mortality, which is 20-fold higher in in individuals aged ≥ 80 compared to 50-59-year-olds [56].

An observational study in the UK analyzed more than 20,000 hospitalized COVID-19 cases. They reported a median age of 73 years, with men being more frequently admitted (60%) than women (40%) [68]. Comorbidities play a central role in COVID-19 disease progression. Over 75% of hospitalized COVID-19 cases had major comorbidities [68]. The most common comorbidity was chronic cardiac disease (30.9%), followed by diabetes (20.7%), non-asthmatic chronic pulmonary disease (17.7%), asthma (14.5%), and chronic kidney disease (16.2%) [68].

#### Respiratory Syncytial Viruses (RSV)

RSV occurs annually and overlaps with seasonal influenza [74]. It is transmitted via respiratory droplets and leads to a spectrum of diseases ranging from rhinorrhea to life-threatening lower respiratory tract infections, bronchiolitis, and pneumonia [75]. The first line of defense against this virus are respiratory epithelial cells, which are also the main target of RSV [76].

Morbidity and mortality for RSV infections are highest in the infants and in immunocompromised or older adults [77, 78]. RSV infection can cause cardiac and pulmonary complications [77]. It is estimated that approximately 177,000 adults are hospitalized annually for RSV infection. In the age group of > 65 years, more than 17,000 deaths are estimated in the US per year [79]. To date, there is no effective therapy available and active vaccines against RSV are under development but have not yet come to market [80, 81].

### Fungal infections

Statistically, fungal respiratory diseases are most prevalent in the elderly and in immunocompromised patients [82]. In particular *Aspergillus*, and in endemic regions, *Cryptococcus*, and *Histoplasma* species manifest themselves in the lungs and can spread systemically in the body, leading to severe courses of disease and even death. However, the cause of increased susceptibility to fungal respiratory disease is not age per se, but rather the host’s immune status [83]. The major risk factor is immunosuppression caused by comorbidities and medications that is increasingly seen in elderly patients. Chemotherapy for the treatment of cancer and hematological malignancies, especially hematopoietic stem cell transplantation (HSCT) and immunosuppressive drugs to prevent graft rejection after solid organ transplantation, can also influence the immune response against fungal infections [84, 85].

Existing lung infections can also be severe risk factors that lead to, for example, severe influenza- and COVID-19-associated pulmonary aspergillosis [86-88]. A multicenter study has shown that 20-30% of critically-ill patients with influenza developed invasive pulmonary aspergillosis (mean age 60 years) resulting in an increased mortality of 40-60% [88].

## 4. Clinical trials on pneumonia and aging

We performed a PubMed search using the keywords “Pneumonia AND Aging” to gain insight into the study landscape regarding clinical trials. A total of 103 hits were recorded during the 10-year period from July 2012 to July 2022. Of these publications, we analyzed a total of 33 as suitable to our search (Supplementary Table 1). Among these studies, a total of 14 clinical trials were focused on COVID-19.

Few studies on pneumonia in the elderly (> 65 years) have been published in the last 10 years, and some had only small sample sizes [89]. In general, studies analyzing mortality rates in CAP and COVID-19 found that patients who died were older than those who survived [90].

Aging individuals can be divided in three groups, group 1 (age 65 to 74), group 2 (75 to 84), and group 3 (over 85 years) [91]. However, our research reveals that from the 33 suitable studies, 10 of them performed their investigations with patients who do not fulfill the criteria of aging.

The age variable is often addressed in the context of a therapeutic trial, where it is particularly noticeable that pharmacokinetic profiles of antibiotic treatment show altered parameters in the elderly and that dosage adjustments have been recommended [92]. The studies we analyzed indicated that vaccination against pneumonia with 13-valent pneumococcal conjugate vaccine (PCV13), and the recently licensed higher-valent pneumococcal vaccines (i.e. PCV15, PCV20, PCV21) is safe and effective in adults 65 years of age and older [93, 94]. Regarding pathogens, it has been published that nursing home residency is not a risk factor for multidrug resistance [95].

## 5. Diagnostic strategies

The diagnosis of pneumonia and other respiratory infections can be challenging due to the similarity of symptoms between different pathogens and the lack of specificity of clinical signs. Several diagnostic methods are available for the detection of respiratory infections, including clinical evaluation, radiological imaging, and laboratory tests. However, each method has its limitations.

Overall, pneumonia is diagnosed clinically according to the CRB-65 index, with the score being a good prediction of lethality risk [96-98]. CRB-65 (C = confusion, R = respiratory rate, B = blood pressure, over 65 years), formed through the determination of three clinical and one anamnestic parameter, is easy to apply in outpatient settings. The following criteria are examined: (I) respiratory rate ≥ 30/min, (II) diastolic blood pressure ≤ 60 mmHg or systolic blood pressure < 90 mmHg, (III) confusion, and (IV) age ≥ 65 years. Nevertheless, CRB-65 has important limitations. It has been shown that in elderly patients, functional status is a prognostic influence and the predictive value of CRB-65 for low lethality in elderly patients and/or nursing home residents is insufficient.

To confirm a diagnosis of community-acquired pneumonia, evidence of a new-onset infiltrate is required based on a radiologic image of the lungs. The diagnosis of pneumonia in the elderly is challenging due to the low sensitivity and specificity of chest radiography (X-rays) [99].

X-rays are a common diagnostic tool used to detect pneumonia, showing the extent of the disease as well as monitoring the treatment response [100]. However, in cases with high clinical suspicion of pneumonia and negative or non-conclusive X-rays, especially in patients with comorbidities, a computed tomography (CT) scan should be performed. For immunocompromised elderly patients in particular, a CT scan should be a first line diagnostic approach, especially if atypical (viral or fungal) pneumonia or pulmonary complications like empyema or abscess are suspected, as the sensitivity of X-rays in this group is low [11, 100].

**Figure 2.**
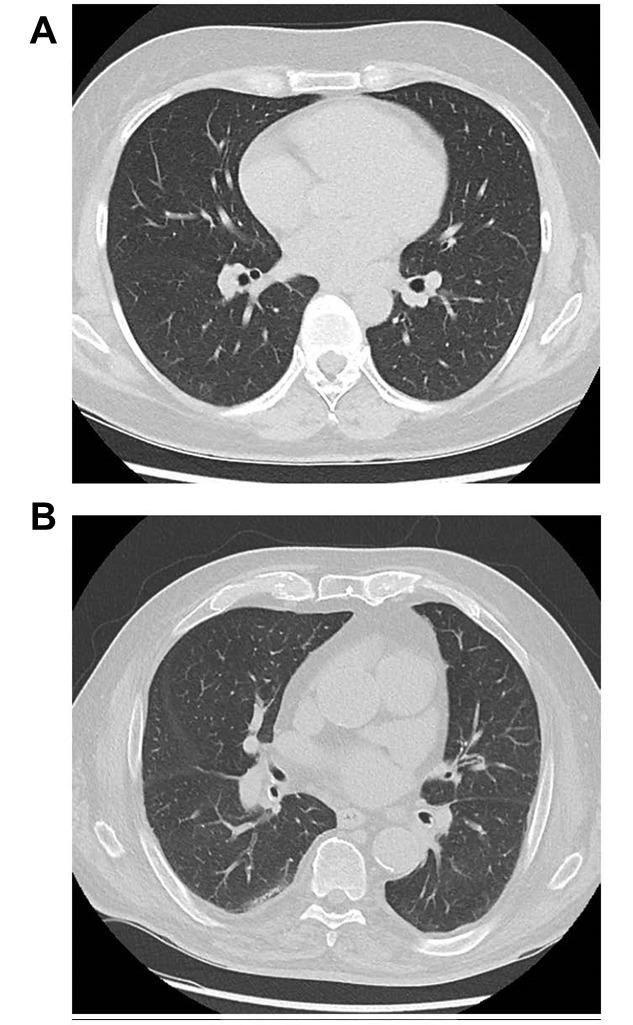
**Computed tomography scan (CT) of the lungs**. (**A**) healthy young individual; (B) discrete subpleural reticulations in the dorsal lung segments of the right lower lobe in an asymptomatic 85-year-old patient.

The morphology of an elderly lung differs from that of a young, healthy individual (Fig. 2). Common age-related changes seen in asymptomatic elderly patients are discreet, predominantly subpleural, basal reticulations, as well as centrilobular emphysema in upper lung zones and progressive calcification of the airways and ribcage [101, 102]. Moreover, many patients show small basal atelectasis due to poor ventilation of lower lung zones, as well as basal effusions [103].

It is worth emphasizing that there is a considerable overlap in the radiological changes caused by different pathogens, therefore the morphological pattern of infectious infiltrates should not be used as an indicator of a specific pathogenic agent [104]. Without clinical information, differentiation between pneumonia and other lung processes causing consolidation is often not possible. Moreover, motion artifacts due to breathing are a serious problem in image interpretation in this age group [102]. Clinical signs are also reported less often in older patients compared to younger ones. Chest pain and fever may occur more rarely in the elderly, whereas hypoxemia is not affected by age [11, 105].

All patients hospitalized for moderate and severe pneumonia require an appropriate microbiological diagnosis. Blood culture, a urine antigen test for *Legionella spp.*, and an adequate sputum sample for the detection of bacteria via Gram-staining and culture is highly recommended. However, diagnosis is mainly limited by the inabilities of elderly patients to produce high-quality expectorate samples, and the implementation of sputum induction is often underused in this situation [106]. In addition, molecular testing via nucleic acid amplification for viruses should be performed if there is adequate epidemiological evidence [107].

Although risk factors for multidrug-resistant organisms are more common in older adults, especially among long-term care facility residents, no substantial changes in the bacterial spectrum of community-acquired pneumonia have occurred in Germany, Austria, and Switzerland in recent years [108, 109]. Therefore, the antibiotic therapy for pneumonia is concocted through risk stratification, and vaccination against influenza viruses/*S. pneumonia*/SARS-CoV-2 represents the most effective preventive strategy [110].

In conclusion, diagnosing respiratory infections in the elderly population can be challenging due to atypical symptoms, the presence of underlying comorbidities, and limitations in diagnostic tests. The management of respiratory infections in the elderly requires careful consideration of antibiotic selection, the potential for adverse drug reactions, and the need to prevent the development of antibiotic resistance. Further research is needed to develop more accurate and rapid diagnostic methods that can improve the management of respiratory infections in the elderly population.

## 6. Therapy and prevention of lung infections in the elderly

### Therapy

The treatment of lung infections in the elderly is not merely a matter of the correct choice of antibiotic therapy, but also needs to address age-specific comorbidities and functional decline in a comprehensive therapeutic effort. Individual risk factors are associated with poor functional recovery in the elderly population, particularly the coexistence of dysphagia, sarcopenia, and aspiration pneumonia [111]. Typical comorbidities in old age, such as Parkinson’s disease or other cognitive deficiencies, may aggravate the course of pneumonia more than age-induced changes of respiratory mechanics per se. Therefore, special attention should be paid to the detection of dysphagia and nutritional support, as well as to adherence to oral drug regimens required to treat underlying comorbidities.

Older adults with chronic diseases often present with an impaired cough sensitivity [112], resulting in microaspiration and increased mortality from pneumonia [113]. Cough efficiency is often severely impaired during an acute episode of a respiratory infection in the elderly and the frail. In this situation, the removal of airway secretions can be of paramount importance. The use of oscillating positive expiratory pressure systems in combination with nebulizers, intensive physiotherapeutic interventions, or (repeated) bronchoscopic removal of airway secretions may be warranted.

Another age-specific aspect concerns the use and adherence to inhalation therapies in patients with obstructive airway diseases, which are common comorbidities in the elderly population. Special attention should be paid to choosing an appropriate device, and to training patients in proper inhalator use [114].

**Figure 3.**
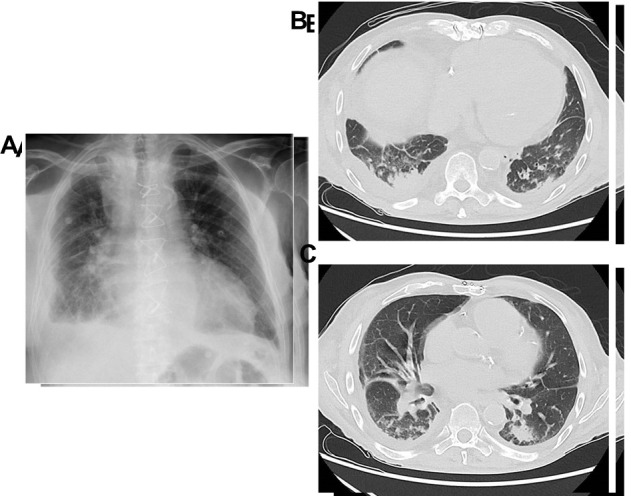
**Elderly patient with a high clinical suspicion of pneumonia**. Because of overlapping pulmonary oedema and effusion, X-ray (A) was non-conclusive. In a subsequently performed CT scan, however, small consolidations in (B, C) right lower lobe are visible.

Furthermore, some additional considerations concern antibiotic treatment in the elderly. It is a well-documented fact that the use of macrolides or fluorochinolones may be associated with an increased risk of sudden cardiac death in at-risk patients [115]. Arrhythmias and impaired cardiac function are among the most frequent comorbidities in old age. Common alternatives are penicillins, which may not always be the best choice. Higher rates of allergies to the β-lactam agent present in penicillin have been reported in older patients [116], who have been frequently exposed to older formulations of penicillin with higher potentials to cause allergic reactions. Older people often have many comorbidities resulting in polypharmacy, which is estimated to reach approximately 7% in the USA alone. Drug interactions are therefore common and need to be avoided (Fig. 3).

### Prevention

Because many elderly people do not fully recover from an acute episode of pneumonia, which in turn might impair daily activities and even result in loss of independence, its prevention is an important measure to ensure healthy aging and to preserve quality of life.

Prevention strategies for pneumonia include vaccination, infection control measures, and management of underlying comorbidities.

The essential preventive measure is vaccination, with vaccination against pneumococci, influenza virus, and SARS-CoV-2 currently recommended. Since the recommendations for SARS-CoV-2 vaccination change very quickly and will most likely be outdated by the time this article is published, only vaccination against influenza and *S. pneumoniae* will be discussed here. In addition, the latest aspects regarding vaccination against RSV will be highlighted.

The WHO recommends influenza vaccination for the prevention of influenza in all elderly persons aged 65 years and older [117]. Because of the high variability of influenza viruses with regard to antigenic shift (i.e. exchange of RNA segments leading to novel strains such as H5N1), leading to pandemics and antigenic drift (point mutations), annual adjustments of seasonal vaccines and annual vaccination is necessary against these viruses. However, the immunogenicity of influenza vaccination is lower in older adults compared to young ones, and factors such as frailty and co-morbidities further decrease vaccine-induced immune responses. In a recent review, pooled vaccine effectiveness against any type of influenza of 51% (95% confidence interval [CI]: 45- 58) for working-age adults and 37% (95 % CI: 30-44) for older adults was calculated. However, the vaccine effectiveness varies from year to year, depending on the antigenic match between the vaccine and the circulating viruses. Optimized vaccines, including adjuvanted and high-dose vaccines, provide better immunogenicity and effectiveness for elderly adults, and hence should replace standard-dose vaccines for the elderly [117].

*S. pneumoniae* is the most common cause of bacterial CAP in adults. Over 90 distinct serotypes based on polysaccharide capsule have been identified to date. However, less than 30 serotypes are responsible for the majority (>90%) of invasive pneumococcal disease. Two types of vaccines are available against *S. pneumoniae*: polysaccharide vaccines covering 23 serotypes (PPV 23), which contain the purified bacterial capsule polysaccharides, and conjugated vaccines (PCV) covering up to 20 serotypes, for which the polysaccharides are conjugated to carrier proteins. The immune response to the PPSV23 vaccine is a T-cell independent immune response only, while the immune response to PCV vaccination is a T-cell dependent response that produces memory B-cells and reduces carriage of the bacteria in the respiratory track. The PPSV23 vaccine does not reduce bacterial carriage and does not produce memory B-cells [118]. Clinical efficacy of the PCV13 vaccine was tested in a large Phase IV randomized, placebo-controlled trial of over 84,000 older adults. In the per-protocol analysis, PCV13 was 45.6% (95% CI: 21.8-62.5%) effective against first episodes of CAP caused by vaccine-type strains, and 75.0% (95% CI: 41.1-90.8%) effective against vaccine-type invasive pneumococcal disease, respectively [93]. For those who have never received any pneumococcal conjugate vaccine, the Centers for Disease Control and Prevention (CDC) recommends PCV15 or PCV20 (pneumococcal conjugate vaccines covering 15 and 20 serotypes, respectively) for adults 65 years or older or for adults 19 through 64 years old with certain medical conditions/risk factors. If PCV15 is used, it should be followed by a dose of PPSV23 to increase coverage [119].

RSV has historically been associated mainly with severe respiratory infections in infants, but older persons, particularly frail ones, are also at high risk for severe disease [80]. In the United States alone, RSV infections in older adults account for approximately 177,000 hospitalizations and 14,000 deaths every year [120]. A growing number of RSV vaccine candidates in different formats (particle-based vaccines, vector-based vaccines, subunit vaccines, and live-attenuated vaccines) are being developed and are now at different stages, many of them already being in the clinical stage [121]. Recently, promising results of a phase 2a study of an intramuscular injection of a bivalent prefusion F (RSVpreF) vaccine in healthy adults (18 to 50 years of age) were reported [122]. The phase 3 trial of a pre-F sub-unit vaccine, RSVpreF (Pfizer), is currently recruiting adults ≥ 60 years (ClinicalTrials.gov: NCT05035212), with trial completion estimated for June 2024.

In addition to vaccination, the best known and best studied pneumonia prevention strategy, it is important to recognize and control modifiable risk factors related to the onset of pneumonia. These factors are often associated with an increased risk of aspiration and include dysphagia and malnutrition, poor oral hygiene, and lifestyle related risk factors such as smoking and alcohol consumption. In addition, a careful review of the prescribed medications and a close monitoring of side-effects can also reduce the risk of pneumonia [123]. For example, an increased risk of pneumonia has been reported with gastric-acid-suppressive drugs (proton-pump inhibitors (PPI) and Histamine 2 receptor antagonists), inhaled corti-costeroids, and antipsychotics [124]. Furthermore, respiratory physiotherapy and mobilization improves the functional status and hence may help reduce the risk of pneumonia.

Infection control measures, such as hand hygiene, respiratory etiquette, and isolation precautions, can also help to prevent pneumonia. These measures are particularly important in healthcare settings, where the risk of healthcare-associated pneumonia is higher.

In addition, the management of underlying comorbidities, such as chronic obstructive pulmonary disease (COPD), heart failure, and diabetes, can also help to prevent pneumonia. By improving the control of these comorbidities, the risk of developing pneumonia can be reduced.

Furthermore, antibody therapies, such as monoclonal antibodies, are also being investigated as a potential prevention approach for pneumonia. These therapies can provide passive immunity against specific pathogens and may be particularly useful in high-risk populations, such as the elderly.

In conclusion, current prevention methods, including vaccination, infection control measures, and management of underlying comorbidities, have been shown to be effective in reducing the incidence of pneumonia in the elderly population. However, vaccine coverage rates remain suboptimal, and new prevention approaches are needed to provide broader protection against pneumonia and other respiratory infections. New vaccines and antibody therapies show promise as potential prevention approaches and have the potential to have a significant impact on the health outcomes of elderly patients.

## 7. Conclusion

In summary, the elderly population is more susceptible to lung infections and tends to experience more severe disease compared to younger individuals. Our review highlights structural changes of the aging lung, the impaired immune system, and the challenging diagnosis of respiratory infection in elderly patients. Microbiological diagnosis shows that the frequency of pathogens did not differ between age groups except for the more frequently in elderly patients’ samples detected *S. pneumoniae* and *H. influenzae*, and the less frequently diagnosed *M. pneumoniae.* The morphology of elderly lungs differs, and computed tomography is preferred as the most appropriate diagnostic imaging technique.

Since only limited clinical studies have examined the effects of aging on pneumonia, further research is needed to elucidate the specific mechanisms that contribute to increased susceptibility and severity of lung infections in the elderly. This research should include investigating the role of the lung microbiome and the interactions between the immune system and comorbidities.

In clinical practice, it is important to prioritize prevention strategies, such as vaccination and management of comorbidities, to reduce the risk of lung infections in the elderly. Additionally, early recognition and treatment of infections are critical to improving health outcomes. Improving health outcomes in the aging population requires a comprehensive approach that addresses both the prevention and treatment of lung infections. This could include vaccination campaigns targeted at the elderly, management of comorbidities, and early recognition and treatment of infections.

Additionally, improving respiratory health through exercise and smoking cessation might contribute to reducing the risk of lung infections in the elderly. These strategies could lead to a reduction in morbidity and mortality from lung infections in this vulnerable population.

## Supplementary Materials

The Supplementary data can be found online at: www.aginganddisease.org/EN/10.14336/AD.2023.0329.
