# Supplementary material for: Respiratory Infections in the Aging Lung: Implications for Diagnosis, Therapy, and Prevention
Source: Aging Dis. 2023 Aug 1;14(4):1091–104. doi: 10.14336/AD.2023.0329 (PMC10389836; doi:10.14336/AD.2023.0329)
Supplement: Supplementary file 1 — The Supplementary data can be found online at: www.aginganddisease.org/EN/10.14336/AD.2023.0329. [file AD-14-4-1091-s.pdf]

SUPPLEMENTARY DATA

Respiratory Infections in the Aging Lung: Implications for Diagnosis, Therapy, and Prevention

Antje Häder, Nilay Köse-Vogel, Luise Schulz, Lucja Mlynska, Franziska Hornung, Stefan Hagel, Ulf Teichgräber, Susanne M. Lang, Mathias W. Pletz, Claude Jourdan Le Saux, Bettina Löffler, Stefanie Deinhardt-Emmer

© 2023. Häder A et al. Published online at https://www.aginganddisease.org/EN/10.14336/AD.2023.0329

SUPPLEMENTARY DATA

Supplementary Table 1. Overview of clinical trials extracted from PubMed between 07/2012 and 07/2022 using the keywords "Pneumonia AND Aging" after exclusion of studies with inappropriate subject matter or studies in which the patients were under the age of 65.

Reference	Title	Content	Summary
Nutrients. 2021 May 2113(6):1760. doi: 10.3390/nu13061760.	*Calcifediol Treatment and Hospital Mortality Due to COVID-19: A Cohort Study*	Drugs/ Therapy	Pneumonia in the elderly population is a major burden for the health care system. Therefore, appropriate treatment strategies are of particular importance and are being investigated in numerous studies. However, therapy effectiveness is lower in the older patient group.
Am J Respir Crit Care Med. 2019 Nov 15200(10):1282-1293. doi: 10.1164/rccm.201812-2328OC.	*Simvastatin Improves Neutrophil Function and Clinical Outcomes in Pneumonia. A Pilot Randomized Controlled Clinical Trial*	Drugs/ Therapy	
J Infect Chemother. 2014 Mar20(3):199-207. doi: 10.1016/j.jiac.2013.10.010. Epub 2013 Dec 11.	*Contradiction between in vitro and clinical outcome: intravenous followed by oral azithromycin therapy demonstrated clinical efficacy in macrolide-resistant pneumococcal pneumonia*	Drugs/ Therapy	
Clin Interv Aging. 2016 Oct 311:1379-1385. doi: 10.2147/CIA.S114515. eCollection 2016.	*Vitamin E administration may decrease the incidence of pneumonia in elderly males*	Drugs/ Therapy	
Geriatr Gerontol Int. 2019 Oct19(10):1006-1009. doi: 10.1111/ggi.13760. Epub 2019 Aug 15.	*Effect of long-term clarithromycin therapy on prevention of pneumonia in older adults: A randomized, controlled trial*	Drugs/ Therapy	
Clin Interv Aging. 2020 Nov 915:2109-2115. doi: 10.2147/CIA.S268140. eCollection 2020.	*Marked Reduction in 28-day Mortality Among Elderly Patients with Severe Community-acquired Pneumonia: Post Hoc Analysis of a Large Randomized Controlled Trial*	Drugs/ Therapy	
J Clin Pharmacol. 2014 Jul54(7):742-52. doi: 10.1002/jcph.265. Epub 2014 Jan 22.	*A series of pharmacokinetic studies of ceftaroline fosamil in select populations: normal subjects, healthy elderly subjects, and subjects with renal impairment or end-stage renal disease requiring hemodialysis*	Drugs/ Therapy	
Drugs Aging. 2017 Feb34(2):115-121. doi: 10.1007/s40266-016-0431-9.	*Meropenem Dosing Based on a Population Pharmacokinetic-Pharmacodynamic Model in Elderly Patients with Infection of the Lower Respiratory Tract*	Drugs/ Therapy	
Medicina (Kaunas). 2021 Oct 957(10):1079. doi: 10.3390/medicina57101079.	*Effect of Vitamin D Supplementation on Muscle Status in Old Patients Recovering from COVID-19 Infection*	Drugs/ Therapy	
J Am Geriatr Soc. 2016 Mar64(3):501-9. doi: 10.1111/jgs.14013.	*Effect of Structured Physical Activity on Respiratory Outcomes in Sedentary Elderly Adults with Mobility Limitations*	Lifestyle intervention/ Therapy	
N Engl J Med. 2021 Sep 23385(13):1172-1183. doi: 10.1056/NEJMoa2107659. Epub 2021 Jun 30.	*Safety and Efficacy of NVX-CoV2373 Covid-19 Vaccine*	Vaccination	Because of the impaired immune system in the elderly, vaccination development for this patient group is a great challenge. Comorbidities in the elderly patient group complicate preventive vaccination strategies.
Blood. 2022 Mar 10139(10):1588-1592. doi: 10.1182/blood.2021014124.	*COVID-19 in vaccinated adult patients with hematological malignancies: preliminary results from EPICOVIDEHA*	Vaccination	
Hum Vaccin Immunother. 201511(7):1825-7. doi: 10.1080/21645515.2015.1043502.	*Prevention of adult pneumococcal pneumonia with the 13-valent pneumococcal conjugate vaccine: CAPiTA, the community-acquired pneumonia immunization trial in adults*	Vaccination	
Sci Immunol. 2021 Nov 126(65):eabk1741. doi:	*Immune signatures underlying post-acute COVID-19 lung sequelae*	Immuno-aging	Aging is associated with a dysregulated immune response

© 2023. Häder A et al. Published online at https://www.aginganddisease.org/EN/10.14336/AD.2023.0329

10.1126/sciimmunol.abk1741. Epub 2021 Nov 12.			caused by various pathomechanisms (impaired neutrophil migration, B-cell response, proinflammation). Older individuals show an increased susceptibility to infections and a reduced response to vaccination. The older immune system is less able to respond to infections and effectively protect the organism. The SARS-CoV-2 pandemic in particular illustrates that the body's response to novel pathogens is age-dependent.
Front Immunol. 2021 Jul 2312:690534. doi: 10.3389/fimmu.2021.690534. eCollection 2021.	*Evolution of Human Memory B Cells From Childhood to Old Age*	Immuno-aging	
Cells. 2021 Nov 3010(12):3373. doi: 10.3390/cells10123373.	*Age Related Differences in Monocyte Subsets and Cytokine Pattern during Acute COVID-19-A Prospective Observational Longitudinal Study*	Immuno-aging	
J Clin Invest. 2021 Jan 4131(1):e142966. doi: 10.1172/JCI142966.	*SARS-CoV-2-specific antibody rearrangements in prepandemic immune repertoires of risk cohorts and patients with COVID-19*	Immuno-aging	
Am J Respir Crit Care Med. 2017 Nov 15196(10):1325-1336. doi: 10.1164/rccm.201704-0814OC.	*Pulmonary Infections in the Elderly Lead to Impaired Neutrophil Targeting, Which Is Improved by Simvastatin*	Immuno-aging	
Mech Ageing Dev. 2021 Jan193:111413. doi: 10.1016/j.mad.2020.111413. Epub 2020 Dec 8.	*Decreased serum levels of the inflammaging marker miR-146a are associated with clinical non-response to tocilizumab in COVID-19 patients*	Immuno-aging	
PLoS One. 2021 Jul 3016(7):e0254261. doi: 10.1371/journal.pone.0254261. eCollection 2021.	*Characteristics of aspiration pneumonia patients in acute care hospitals: A multicenter, retrospective survey in Northern Japan*	Aspiration pneumonia	Aspiration pneumonia occurs more frequently in old age and is associated with longer fasting periods, longer hospitalization, and higher in-hospital mortality.
Clin Rehabil. 2017 Aug31(8):1049-1056. doi: 10.1177/0269215516673208. Epub 2016 Oct 14.	*Independent exercise for glottal incompetence to improve vocal problems and prevent aspiration pneumonia in the elderly: a randomized controlled trial*	Aspiration pneumonia	
Thromb Haemost. 2022 Feb122(2):257-266. doi: 10.1055/a-1692-9939. Epub 2021 Nov 10.	Comparison of Thrombotic Events and Mortality in Patients with Community-Acquired Pneumonia and COVID-19: A Multicenter Observational Study	Case mortality	High mortality rate of CAP patients is associated to old age.
PLoS One. 2018 Jan 513(1):e0190716. doi: 10.1371/journal.pone.0190716. eCollection 2018.	Naso- and oropharyngeal bacterial carriage in nursing home residents: Impact of multimorbidity and functional impairment	Pathogens	Long-term carriage of *S. pneumoniae* and MDR bacteria is rare.

© 2023. Häder A et al. Published online at https://www.aginganddisease.org/EN/10.14336/AD.2023.0329
